# The most effective exercise to prevent obesity: A longitudinal study of 33,731 Taiwan biobank participants

**DOI:** 10.3389/fnut.2022.944028

**Published:** 2022-09-23

**Authors:** Wan-Yu Lin

**Affiliations:** ^1^Institute of Epidemiology and Preventive Medicine, College of Public Health, National Taiwan University, Taipei, Taiwan; ^2^Master of Public Health Degree Program, College of Public Health, National Taiwan University, Taipei, Taiwan; ^3^Department of Public Health, College of Public Health, National Taiwan University, Taipei, Taiwan

**Keywords:** body fat percentage, body mass index, hip circumference, waist circumference, waist-hip ratio

## Abstract

Regular physical exercise is recommended to reduce the risk of obesity. However, it remains unclear which activities are more effective in preventing obesity. In this study, five obesity indices and lifestyle factors of 33,731 Taiwan Biobank adults were measured/collected twice with a mean time interval of 4.06 years. A linear mixed effects model was fitted to assess the associations of exercises with obesity indices, in which a random intercept term was used to account for individual differences. The five obesity indices included body mass index (BMI), body fat percentage (BFP), waist circumference (WC), hip circumference (HC), and waist-hip ratio (WHR). Among 23 exercises, jogging and yoga were consistently the most effective choices across all five obesity indices. One more weekly hour to jog was associated with a 0.093 kg/m^2^ decrease in BMI (*p* = 4.2E-20), a 0.297% decrease in BFP (*p* = 3.8E-36), a 0.398 cm decrease in WC (*p* = 1.6E-21), and a 2.9E-3 decrease in WHR (*p* = 1.3E-17). One more weekly hour to perform yoga was associated with a 0.225 cm decrease in HC (*p* = 6.4E-14). Jogging is an exercise for the entire body. Arms swing, waist turn, legs and feet run, and shoulders and abdomen are also involved in this act. By contrast, many yoga poses use muscles around the hips and pelvis, and therefore yoga is the most effective exercise to reduce HC.

## Introduction

Obesity is associated with an increased risk of metabolic syndromes, causing severe health problems worldwide (1). The prevalence and disease burden of obesity have grown rapidly, highlighting the importance of obesity prevention. Performing regular physical exercise has been recommended to prevent obesity (2–6). However, it remains unknown which activities are more effective in lowering the risk of obesity.

Exercises can be roughly categorized as “aerobic exercise” and “anaerobic exercise.” Aerobic exercise was defined as any continuous and rhythmic activity that involves large muscle groups, according to the American College of Sports Medicine (ACSM) (7, 8). Examples of aerobic exercise include jogging, brisk walking, swimming, mountain climbing, dancing, cycling, etc. During aerobic exercises, the heart beats faster to supply more oxygen and blood to the working muscles. The ACSM defined the anaerobic exercise as any intense physical activity of short duration, fueled by the energy sources in the contracting muscles rather than the inhaled oxygen (9). Examples of anaerobic exercise include resistance exercise, strength training, and weight training.

Many randomized controlled trials (RCTs) have investigated the efficacy of exercise in preventing obesity. Banz et al. investigated the effects of aerobic exercise (sample size of this group, *n* = 14) and resistance exercise (*n* = 12) on body fat percentage (BFP), and waist-hip ratio (WHR) (10). Both groups demonstrated modest decreases in WHR after 10 weeks of training, but only the group performing resistance exercise showed a reduction in BFP.

Another 10-month RCT showed supervised aerobic exercise resulted in a significant weight loss for 141 overweight and obese participants (11). Moreover, a 3-month RCT also found that aerobic exercise (*n* = 20) reduced more weight in obese subjects than anaerobic exercise (*n* = 20) (12).

Among a variety of exercises, yoga has caught much attention. Originated in India more than 5,000 years ago, yoga is an act of harmonizing and balancing the human mind, body, and emotions. Yoga is usually not considered an aerobic exercise, because the mind is relaxed and the heartbeat is normal during yoga practices (13). Some RCTs have focused on the effect of performing yoga to prevent obesity. For example, yoga training for 14 weeks has been found to reduce male obesity, based on an RCT with a sample size of 72, in which 37 males were in the yoga group and 35 were in the control group (14).

More than staying fit, performing yoga can also help manage complex diseases. People practicing yoga are usually encouraged to follow a yogic diet. Advised by Yogi Bhajan, the yogic diet suggests people eat whole and unprocessed foods such as fresh vegetables, grains, and fruits. Practicing yoga regularly and following the yogic diet can help control glycemic levels and reduce the risk of type 2 diabetes (15).

Although RCTs can quantify the effects of performing exercises on reducing obesity indices, they are usually small to medium regarding sample sizes. Moreover, the investigated activities are typically limited to two or three kinds (10–12, 14, 16). By contrast, large-scale observational studies can afford to compare the effects of various exercises on obesity.

Recently, Lin et al. investigated whether 18 kinds of self-reported regular exercise are associated with an attenuation of genetic susceptibility to obesity (17). A total of five obesity indices were analyzed, including body mass index (BMI), BFP, waist circumference (WC), hip circumference (HC), and WHR. Among the 18 exercises, jogging consistently exhibited the most substantial evidence to attenuate the genetic effects on the five obesity indices. However, that study focused on gene-exercise interactions rather than an even more critical issue–which exercises effectively prevent obesity.

Observational studies are usually criticized for not performing randomization on individuals, and therefore controlling for individual differences seems impossible. We applied for the longitudinal data from the Taiwan Biobank (TWB) to address this issue. A subject-specific random effect to account for individual differences was incorporated into our linear mixed effects model. As a result, we evaluated the associations of time to perform 23 exercises with the five obesity indices while controlling individual differences.

## Materials and methods

### Taiwan biobank

Since October 2012, TWB has recruited Taiwan residents aged 30 to 70 years and collected their genomic and lifestyle information (18). After signing informed consent, community-based volunteers took physical examinations and provided their blood and urine samples. TWB researchers further collected lifestyle factors through a face-to-face interview with each participant.

As of February 2021, 33,731 individuals have been surveyed twice with a mean time interval of 4.06 years (standard deviation, s.d. = 1.23 years). Five obesity indices were measured, and lifestyle factors (including the exercise information) were collected for these 33,731 adults in the baseline and follow-up surveys.

### Five obesity indices

We analyzed five obesity-related phenotypes: BMI, BFP, WC, HC, and WHR. BMI is a commonly-used obesity index that can be conveniently calculated by weight and height. BFP measures the percentage of an individual’s body that is composed of fat. WC and WHR are indicators of abdominal obesity (19). HC is one of body size measurements for predicting type 2 diabetes (20).

Body mass index (BMI) was calculated as weight in kilograms over squared height in meters, where body weight and height were measured by TWB researchers. BFP was quantified by bioelectrical impedance analysis using a Tanita body composition analyzer BC-420MA (Tanita Corp., Tokyo, Japan). Following the World Health Organization (WHO) recommendation, WC is the circumference of the midpoint between the iliac crest and lowest rib (21). HC was the largest circumference around the buttocks in a standing position. A non-elastic tape measured both WC and HC. WHR was dimensionless calculated as the ratio of WC to HC.

### Covariates adjusted in all models

In this study, covariates adjusted in all regression models included sex, age (in years), smoking status (yes vs. no), drinking status (yes vs. no), educational attainment (a value ranging from 1 to 7), and the first ten ancestry PCs. In TWB, drinking was defined as a subject having a weekly intake of more than 150 mL of alcohol for at least 6 months and having not stopped drinking when his/her phenotypes were measured. Smoking was defined as a subject who had smoked for at least 6 months and had not quit smoking when his/her phenotypes were measured.

The educational attainment of each subject was surveyed through a face-to-face interview with TWB researchers. It was recorded as a number ranging from 1 to 7, with (1) indicating “illiterate,” (2) “no formal education but literate,” (3) “primary school graduate,” (4) “junior high school graduate,” (5) “senior high school graduate,” (6) “college graduate,” and (7) “Master’s or higher degree.”

Regular exercise was defined as performing 30 min of “exercise” three times a week. “Exercise” includes leisure-time activities such as swimming, cycling, jogging, etc. Individuals with regular exercise would then be asked what kinds of exercise they usually engaged in during the latest 3 months. To prevent a long TWB interview, they were allowed to list at most three kinds of exercise and the frequency and duration when performing each exercise.

### Statistical analysis

To investigate the associations of weekly hours to perform 23 physical exercises with BMI, we considered the following linear mixed effects model:


(1) BMI=i,jβ+0ΣβEkk=123Ek,i,j+ΣβCvv=15

 Covariate+v,i,jγ+iε.i,j

A total of five covariates were adjusted in the model, including sex (male vs. female), age (in years), drinking status (yes vs. no), smoking status (yes vs. no), and educational attainment (an integer from 1 to 7). In model (1), the subscript *i* denotes the *i*th individual, and the subscript *j* represents the *j*th survey, where *i* = 1, …, 33731 and *j* = 1 or 2. *E*_*k*,*i*,*j*_ indicates the weekly hours to perform the *k*th exercise for the *i*th individual at the *j*th survey, where *k* = 1, …, 23. γ_*i*_ is the subject-specific random effect of the *i*th individual, and ϵ_*i,j*_ is the random error term for the *i*th individual at the *j*th survey. The response variable in model (1) can be replaced with the other four obesity indices accordingly.

Model (1) can simultaneously adjust for the effects of the 23 exercises, because an individual may engage in multiple physical exercises (each individual was allowed to report three kinds of exercises, and the frequency and duration when performing each kind of exercise). Moreover, this model incorporated subject-specific random effects γ_*i*_, which accounted for individual differences in obesity indices. The R (version 4.1.1) package “lmerTest” (Tests in Linear Mixed Effects Models; version: 3.1-3) (22) was used to calculate the *p*-value of testing *H*_0_:β_*E*__*k*_, = 0*vs*.*H*_1_:β_*E*__*k*_≠ 0, where *k* = 1, …, 23. According to the Bonferroni correction, with 23 exercises and five obesity indices, *p* < 0.0004 = 0.05/(23×5) was claimed to be significant.

## Results

### Basic characteristics of the 33,731 Taiwan biobank participants

Among the 33,731 TWB participants, 12,058 (35.7%) were males and 21,673 (64.3%) were females. Table 1 presents the basic characteristics of the 33,731 TWB subjects in the two surveys, respectively. The mean age of the 33,731 adults was 51 (s.d. = 10) years in the 1st survey and 55 (s.d. = 10) years in the 2nd survey. The average time interval between the two surveys was 4 (s.d. = 1) years.

**TABLE 1:** Basic characteristics of the 33,731 Taiwan biobank subjects.

	The 1st survey	The 2nd survey	*P*-value^1^
Total, *n*	33,731		—
Males, *n* (%)	12,058 (35.7%)		—
Age (years), mean (s.d.)	51.08 (10.40)	55.14 (10.34)	0
Age range (years)	30∼70	32∼79	
Smoking, *n* (%)	2,682 (8.0%)	2,524 (7.5%)	1.5E-08
Drinking, *n* (%)	1,925 (5.7%)	2,358 (7.0%)	1.2E-31
Regular exercise, *n* (%)	15,287 (45.3%)	16,085 (47.7%)	6.0E-19
Educational attainment, mean (s.d.)^2^	5.34 (1.00)	5.36 (1.01)	2.2E-63
BMI (kg/m^2^), mean (s.d.)	24.1 (3.6)	24.3 (3.7)	9.3E-297
Body fat %, mean (s.d.)	28.5 (7.3)	28.8 (7.5)	2.0E-84
Waist circumference (cm), mean (s.d.)	83.1 (9.8)	84.0 (10.0)	8.3E-181
Hip circumference (cm), mean (s.d.)	95.6 (6.8)	95.7 (7.0)	0.223
Waist-hip ratio, mean (s.d.)	0.87 (0.07)	0.88 (0.07)	5.6E-241

^1^The *p*-value of testing the difference between the two surveys, according to McNemar’s test (for smoking, drinking, and regular exercise) or the paired *t*-test (for age, educational attainment, and the five obesity indices).

^2^Educational attainment was recorded as a number ranging from 1 to 7, with (1) indicating “illiterate,” (2) “no formal education but literate,” (3) “primary school graduate,” (4) “junior high school graduate,” (5) “senior high school graduate,” (6) “college graduate,” and (7) “Master’s or higher degree”.

The percentage of regular exercise increased from 45.3% in the 1st survey to 47.7% in the 2nd survey (the McNemar’s test *p*-value = 6.0E-19). The percentage of smoking was decreased from 8.0 to 7.5% (*p* = 1.5E-8), and that of drinking was increased from 5.7 to 7.0% (*p* = 1.2E-31). Except for HC, all obesity indices significantly increased from the 1st survey to the 2nd survey (paired *t*-test *p*-value ≤ 2.0E-84).

### Associations of weekly hours to perform exercises with obesity indices

**FIGURE 1:**
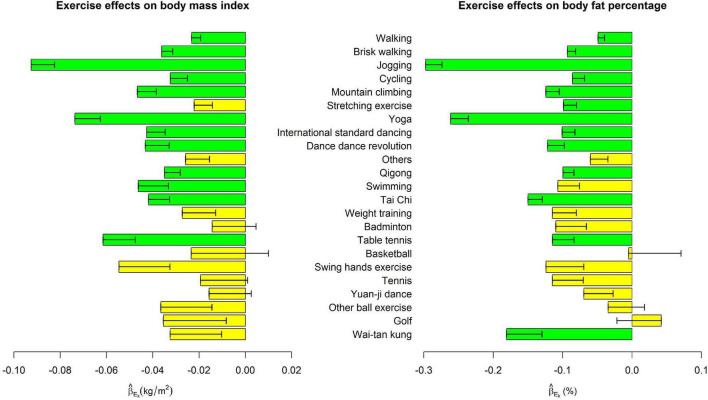
Exercise main effects on body mass index and body fat percentage. The bars represent ${\hat{\beta }}_{E_{k}}$ in model (1), and the black segments mark the standard error of ${\hat{\beta }}_{E_{k}}$, where *k* = 1, …, 23. Bars with *p* < 0.0004 = 0.05/(23 × 5) were highlighted in green color.

**FIGURE 2:**
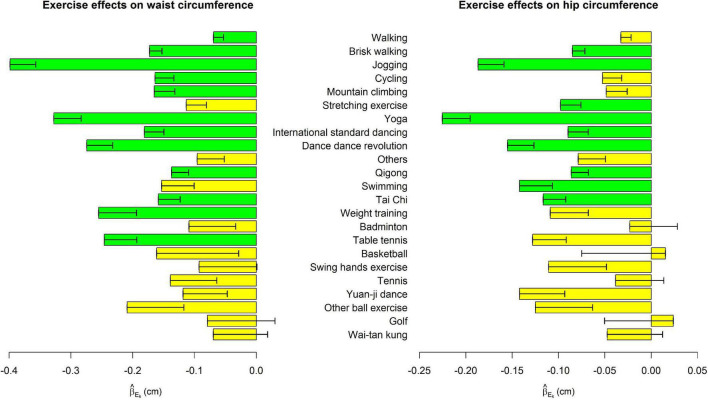
Exercise main effects on waist circumference and hip circumference. The bars represent ${\hat{\beta }}_{E_{k}}$ in model (1), and the black segments mark the standard error of ${\hat{\beta }}_{E_{k}}$, where *k* = 1, …, 23. Bars with *p* < 0.0004 = 0.05/(23 × 5) were highlighted in green color.

**FIGURE 3:**
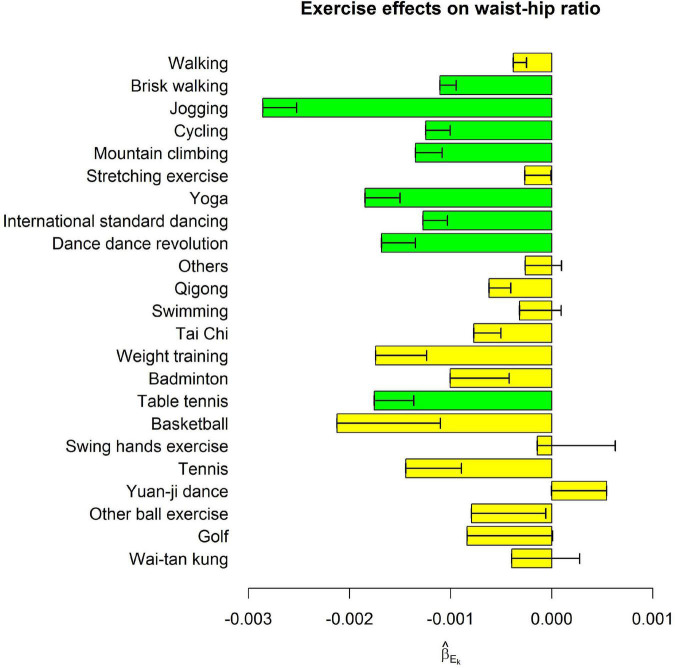
Exercise main effects on waist-hip ratio. The bars represent ${\hat{\beta }}_{E_{k}}$ in model (1), and the black segments mark the standard error of ${\hat{\beta }}_{E_{k}}$, where *k* = 1, …, 23. Bars with *p* < 0.0004 = 0.05/(23 × 5) were highlighted in green color.

Table 2 shows the associations of weekly hours to perform each exercise with five obesity indices, respectively, whereas the effect sizes sizes (β_*E_k*_) in model (1),where k=1, …, 23) were presented in Figures 1–3.

**TABLE 2:** Associations of weekly hours to perform 23 physical exercises with five obesity indices.

	No. of individuals		Mean weekly hours to perform the exercise		BMI (kg/m^2^)		Body fat %		Waist circumference (cm)		Hip circumference (cm)		Waist-hip ratio	
							
	The 1st survey	The 2nd survey	The 1st survey	The 2nd survey	${\hat{\beta }}_{E_{k}}$	*P*-value	${\hat{\beta }}_{E_{k}}$	*P*-value	${\hat{\beta }}_{E_{k}}$	*P*-value	${\hat{\beta }}_{E_{k}}$	*P*-value	${\hat{\beta }}_{E_{k}}$	*P*-value
Walking	5,503	6,363	3.5	3.4	−0.023	4.5E-09	−0.049	2.1E-07	−0.069	2.5E-05	−0.033	3.1E-03	−3.8E-04	3.8E-03
Brisk walking	3,134	3,706	3.5	3.7	−0.036	1.1E-13	−0.093	1.4E-15	−0.173	1.1E-17	−0.085	3.6E-10	−1.1E-03	6.0E-12
Jogging	1,576	1,478	2.4	2.4	−0.093	4.2E-20	−0.297	3.8E-36	−0.398	1.6E-21	−0.187	3.0E-11	−2.9E-03	1.3E-17
Cycling	1,699	1,239	3.3	3.2	−0.032	1.3E-05	−0.086	1.1E-06	−0.164	9.1E-08	−0.053	1.1E-02	−1.2E-03	3.0E-07
Mountain climbing	1,305	1,537	3.1	3.0	−0.047	1.2E-08	−0.124	1.1E-10	−0.165	8.5E-07	−0.049	3.2E-02	−1.4E-03	3.4E-07
Stretching exercise	1,471	1,440	3.6	3.4	−0.022	5.4E-03	−0.099	1.5E-07	−0.113	5.2E-04	−0.098	8.8E-06	−2.7E-04	3.0E-01
Yoga	931	1,070	3.2	2.9	−0.074	2.0E-11	−0.262	2.9E-24	−0.328	1.6E-13	−0.225	6.4E-14	−1.8E-03	9.5E-08
International standard dancing	1,162	1,238	4.5	4.3	−0.043	9.2E-08	−0.101	5.9E-08	−0.181	1.1E-08	−0.090	3.2E-05	−1.3E-03	1.6E-07
Dance dance revolution	909	958	3.3	3.2	−0.043	2.8E-05	−0.122	5.0E-07	−0.274	8.1E-11	−0.155	5.4E-08	−1.7E-03	4.4E-07
Others	521	802	3.1	3.2	−0.026	1.2E-02	−0.060	1.8E-02	−0.096	2.9E-02	−0.079	7.2E-03	−2.6E-04	4.6E-01
Qigong	1,016	1,165	5.0	5.1	−0.035	2.7E-07	−0.100	3.3E-10	−0.137	6.7E-07	−0.086	3.5E-06	−6.2E-04	3.9E-03
Swimming	798	659	2.9	2.9	−0.046	3.6E-04	−0.107	5.7E-04	−0.153	3.6E-03	−0.142	6.6E-05	−3.2E-04	4.4E-01
Tai Chi	875	791	4.8	4.7	−0.042	4.3E-06	−0.150	5.2E-13	−0.159	8.5E-06	−0.117	1.6E-06	−7.7E-04	4.2E-03
Weight training	291	550	2.9	2.6	−0.027	5.9E-02	−0.115	8.8E-04	−0.255	3.2E-05	−0.109	7.9E-03	−1.7E-03	5.5E-04
Badminton	297	270	3.5	3.5	−0.014	4.5E-01	−0.110	1.3E-02	−0.109	1.5E-01	−0.023	6.5E-01	−1.0E-03	8.6E-02
Table tennis	299	326	5.3	5.3	−0.062	9.6E-06	−0.115	2.2E-04	−0.246	3.3E-06	−0.128	4.3E-04	−1.8E-03	7.5E-06
Basketball	123	102	3.0	2.8	−0.023	4.8E-01	−0.005	9.5E-01	−0.161	2.3E-01	0.015	8.6E-01	−2.1E-03	3.8E-02
Swing hands exercise	193	202	2.9	2.8	−0.055	1.3E-02	−0.124	2.3E-02	−0.092	3.2E-01	−0.111	7.6E-02	−1.4E-04	8.5E-01
Tennis	191	175	5.4	5.1	−0.019	3.4E-01	−0.115	9.7E-03	−0.139	6.5E-02	−0.038	4.6E-01	−1.4E-03	8.4E-03
Yuan-ji dance	225	147	5.1	5.1	−0.016	3.9E-01	−0.070	9.8E-02	−0.119	9.8E-02	−0.142	3.6E-03	5.4E-04	3.2E-01
Other ball exercise	114	90	4.2	4.3	−0.037	9.9E-02	−0.035	5.1E-01	−0.209	2.3E-02	−0.125	4.3E-02	−8.0E-04	2.8E-01
Golf	105	89	3.6	4.0	−0.036	1.9E-01	0.042	5.1E-01	−0.079	4.7E-01	0.024	7.5E-01	−8.4E-04	3.2E-01
Wai-tan kung	123	107	5.4	5.2	−0.033	1.4E-01	−0.181	3.8E-04	−0.070	4.3E-01	−0.048	4.3E-01	−4.0E-04	5.5E-01

Among the 23 exercises, jogging, and yoga were consistently the most effective exercises for all five obesity indices. For all indices except HC, jogging was the most effective exercise as far as its effect sizes were concerned. One more weekly hour to jog was associated with 0.093 kg/m^2^ decrease in BMI (*p* = 4.2E-20), 0.297 % decrease in BFP (*p* = 3.8E-36), 0.398 cm decrease in WC (*p* = 1.6E-21), and 2.9E-3 decrease in WHR (*p* = 1.3E-17). Yoga was ranked as the 2nd effective exercise for these four obesity indices. One more weekly hour to perform yoga was associated with 0.074 kg/m^2^ decrease in BMI (*p* = 2.0E-11), 0.262 % decrease in BFP (*p* = 2.9E-24), 0.328 cm decrease in WC (*p* = 1.6E-13), and 1.8E-3 decrease in WHR (*p* = 9.5E-8).

For HC, one more weekly hour to perform yoga was associated with a 0.225 cm decrease in HC (*p* = 6.4E-14). Jogging was ranked as the 2nd effective exercise regarding its effect size on HC. One more weekly hour to jog was associated with a 0.187 cm decrease in HC (*p* = 3.0E-11).

Figures 1–3 show that spending time performing exercise is generally associated with lower obesity indices. Among the 23 kinds of exercise, jogging was associated with the most considerable reduction in four obesity indices and the second-largest decrease in HC. Performing yoga was associated with the largest reduction in HC and the second-largest reduction in the other four obesity indices. Playing table tennis exhibited similar effectiveness to performing yoga in reducing WHR. One more weekly hour to play table tennis was associated with a 1.8E-3 decrease in WHR (*p* = 7.5E-6, not as significant as performing yoga due to fewer people playing table tennis). Because waist turning is frequently required when playing table tennis, this exercise is associated with a noticeable reduction in WHR.

## Discussion

Because diet information was not collected in the TWB, our regression model (1) cannot adjust for this covariate. However, many yoga classes promote a yogic diet in addition to the physical practice of yoga. People performing yoga may also follow the yogic diet (23). The yogic diet suggests people eat whole and unprocessed foods such as fresh vegetables, grains, and fruits (15). Yoga enhances people’s discipline in physical activity and selection of foods (24). Therefore, the effect of “practicing yoga” may be contributed by both performing yoga physically and following the yogic diet.

Moreover, studies have suggested that yoga is associated with weight loss and maintenance (25). Engaging in yoga increases energy expenditure and reduces stress, anxiety, and depression (26). An RCT has found a yoga training for 14 weeks can reduce obesity (14). In addition to staying fit, people performing yoga are usually encouraged to follow a yogic diet. Eating whole and unprocessed foods can help glycemic control (15).

A 12-week yoga intervention reduced obesity measures, including BMI, BFP, WC, and WHR (HC was not served as an outcome measure in that study) (27). Moreover, yoga increased self-esteem and decreased perceived stress (27). Yoga is an exercise to harmonize and balance the human mind, body, and emotions. Yoga is a lifestyle and not merely a kind of exercise (28). Although yoga is not considered an aerobic exercise (13), these factors contribute to the results shown here–yoga is among the most effective exercises to prevent obesity.

Popular yoga poses in Taiwan include “hip-opening yoga poses,” “twist yoga poses,” and “forward bend yoga poses.” “Hip-opening yoga poses” help people stretch their hips and pelvis areas. “Twist yoga poses” help relax the spine and internal organs. “Forward bend yoga poses” loosen the muscles around the human lower back and hips. Many yoga poses involve muscles around the hips and pelvis.

Randomized controlled trials (RCTs) can gauge the efficacy of performing exercise in preventing obesity. However, RCTs are usually small to medium in sample sizes. For example, 312 papers about RCTs of yoga were published from 1975 to 2014. The median study sample size was 59, with a range of 8∼410 and an interquartile range of 31∼93 (29). We included a much larger sample size here than RCTs of physical exercise. A total of 931 and 1,070 TWB individuals practiced yoga regularly in the 1st and 2nd surveys, respectively (Table 2).

Moreover, most RCTs can only afford to compare the effects of two or three exercises (16). For example, a previous study found that performing yoga showed a more significant reduction in BMI than walking (30). Our results are in line with their findings. Moreover, our work involves a comparison of more than 20 exercises.

This is an observational study with long-term follow-up. However, our analysis did not adjust for diet information because it was not collected by the TWB. All the results herein were explained as association signals. Although individuals were not randomized into different exercise groups in this observational study, we incorporated data at two-time points to account for individual differences in obesity indices. Our results are consistent across various obesity indices–jogging and yoga are the two most effective exercises among the 23 exercises.

Our results are reasonable. For example, jogging, brisk walking, and walking are moving on land at different rates. Jogging is an act of moving faster than brisk walking, and then walking. As shown in Figures 1–3, the reduction sizes in the five obesity indices consistently followed this order (jogging > brisk walking > walking). Walking is a relatively mild exercise, and thus, its association with a reduction of obesity indices is not notable.

Jogging and yoga are the two most effective exercises to prevent obesity. This is a consistent conclusion across the five obesity indices. Because many yoga poses involve muscles around the hips and pelvis, yoga is the most effective in reducing HC. On the other hand, jogging is an exercise for the entire body. Arms swing, waist turn, legs and feet run, and shoulders and abdomen are also involved in the act. Jogging outperformed other exercises in four obesity indices: BMI, BFP, WC, and WHR.

Our results showed that one more weekly hour to jog was associated with 0.093 kg/m^2^ decrease in BMI, 0.297% decrease in BFP, 0.398 cm decrease in WC, and 2.9E-3 decrease in WHR. Compared with people without regular exercise, individuals jogging 3 hours per week had lower BMI by ∼0.3 kg/m^2^, lower BFP by ∼0.9%, shorter WC by ∼1.2 cm, and smaller WHR by ∼0.01. These reductions in general obesity indicators (BMI and BFP) and abdominal obesity indicators (WC and WHR) can decrease the risk of cardiovascular diseases (31) and chronic diseases (32). Many studies indicated that the burden of chronic diseases is mainly contributed by overweight and obesity (32–37). Even a slight reduction in population-averaged BMI can significantly decrease the chronic disease burden (32).

## Data availability statement

The original contributions presented in this study are included in the article/supplementary material, further inquiries can be directed to the corresponding author.

## Ethics statement

The studies involving human participants were reviewed and approved by National Taiwan University Hospital (NTUH-REC no. 201805050RINB). The patients/participants provided their written informed consent to participate in this study.

## Author contributions

W-YL conceived the study design, applied for the Taiwan Biobank data, developed the analysis tool, performed the analyses, interpreted the analysis results, wrote the manuscript, and approved the submitted version.
